# Circulating metabolic biomarkers of renal function in diabetic and non-diabetic populations

**DOI:** 10.1038/s41598-018-33507-7

**Published:** 2018-10-15

**Authors:** Clara Barrios, Jonas Zierer, Peter Würtz, Toomas Haller, Andres Metspalu, Christian Gieger, Barbara Thorand, Christa Meisinger, Melanie Waldenberger, Olli Raitakari, Terho Lehtimäki, Sol Otero, Eva Rodríguez, Juan Pedro-Botet, Mika Kähönen, Mika Ala-Korpela, Gabi Kastenmüller, Tim D. Spector, Julio Pascual, Cristina Menni

**Affiliations:** 1 Department for Twin Research, King’s College London, London, UK; 2 Department of Nephrology, Hospital del Mar, Institut Mar d’Investigacions Mediques, Barcelona, Spain; 3 Institute of Bioinformatics and Systems Biology, Helmholtz Zentrum München - German Research Center for Environmental Health, Neuherberg, Germany; 4 Research Programs Unit, Diabetes and Obesity, University of Helsinki, Helsinki, Finland; 5 Nightingale Health Ltd, Helsinki, Finland; 6 Estonian Genome Center, University of Tartu, Tartu, Estonia; 7 Institute of Epidemiology II, Helmholtz Zentrum München - German Research Center for Environmental Health, Neuherberg, Germany; 8 Research Unit Molecular Epidemiology, Helmholtz Zentrum München - German Research Center for Environmental Health, Neuherberg, Germany; 9 Chair of Epidemiology, Ludwig-Maximilians-Universität München, UNIKA-T, Augsburg, Germany; 10 Research Centre of Applied and Preventive Cardiovascular Medicine, University of Turku, Turku, Finland; 11 Department of Clinical Physiology and Nuclear Medicine, Turku University Hospital, Turku, Finland; 12 Department of Clinical Chemistry, Fimlab Laboratories and Finnish Cardiovascular Research Center-Tampere, Faculty of Medicine and Life Sciences, University of Tampere, Tampere, Finland; 13 Department of Nephrology, Consorci Sanitari del Garraf, Barcelona, Spain; 14 Department of Endocrinology and Nutrition, Hospital del Mar, Institut Mar d’Investigacions Mediques, Barcelona, Spain; 15 Department of Clinical Physiology, University of Tampere and Tampere University Hospital, Tampere, Finland; 16 Systems Epidemiology, Baker Heart and Diabetes Institute, Melbourne, Victoria Australia; 17 Population Health Science, Bristol Medical School, University of Bristol, Bristol, UK; 18 Medical Research Council Integrative Epidemiology Unit at the University of Bristol, Bristol, UK; 19 Computational Medicine, Faculty of Medicine, University of Oulu and Biocenter Oulu, Oulu, Finland; 20 NMR Metabolomics Laboratory, School of Pharmacy, University of Eastern Finland, Kuopio, Finland; 21 Department of Epidemiology and Preventive Medicine, School of Public Health and Preventive Medicine, Faculty of Medicine, Nursing and Health Sciences, The Alfred Hospital, Monash University, Melbourne, Victoria Australia; 22 Present Address: Weill Cornell Medical College, New York City, USA

## Abstract

Using targeted NMR spectroscopy of 227 fasting serum metabolic traits, we searched for novel metabolic signatures of renal function in 926 type 2 diabetics (T2D) and 4838 non-diabetic individuals from four independent cohorts. We furthermore investigated longitudinal changes of metabolic measures and renal function and associations with other T2D microvascular complications. 142 traits correlated with glomerular filtration rate (eGFR) after adjusting for confounders and multiple testing: 59 in diabetics, 109 in non-diabetics with 26 overlapping. The amino acids glycine and phenylalanine and the energy metabolites citrate and glycerol were negatively associated with eGFR in all the cohorts, while alanine, valine and pyruvate depicted opposite association in diabetics (positive) and non-diabetics (negative). Moreover, in all cohorts, the triglyceride content of different lipoprotein subclasses showed a negative association with eGFR, while cholesterol, cholesterol esters (CE), and phospholipids in HDL were associated with better renal function. In contrast, phospholipids and CEs in LDL showed positive associations with eGFR only in T2D, while phospholipid content in HDL was positively associated with eGFR both cross-sectionally and longitudinally only in non-diabetics. In conclusion, we provide a wide list of kidney function–associated metabolic traits and identified novel metabolic differences between diabetic and non-diabetic kidney disease.

## Introduction

Chronic kidney disease (CKD) is a major public health problem affecting more than 10% of the population in Western countries^1^, leading to increased cardiovascular (CV) morbidity and mortality^2^. The renal microvascular complication of diabetes (DKD) is the leading cause of end-stage renal disease (ESRD). Despite the efforts in early diagnosis and therapeutic interventions in diabetes control, the rate of ESRD caused by DKD decreases less than the rates of all other diabetes complications^3^.

In recent years, several studies investigated metabolic profiles associated with renal function^4,5^ in the general population^6^ and in type 1 diabetic (T1D) patients^7–9^ to identify biomarkers for disease progression^8^ and mortality^10^. Other studies have looked for metabolic markers of type 2 diabetic (T2D) kidney disease, but sample sizes were small, they lacked independent replication^11^ or were performed in experimental animal models^12^.

Here, we used targeted nuclear magnetic resonance (NMR) spectroscopy to investigate metabolic signatures of renal function in T2D and non-diabetic individuals, combining four European cohorts. Additionally, to gain insights in potential mechanisms of the cross-sectional associations, we investigated longitudinal changes of metabolite levels and renal function and associations with other microvascular complications of T2D.

## Results

**Figure 1:**
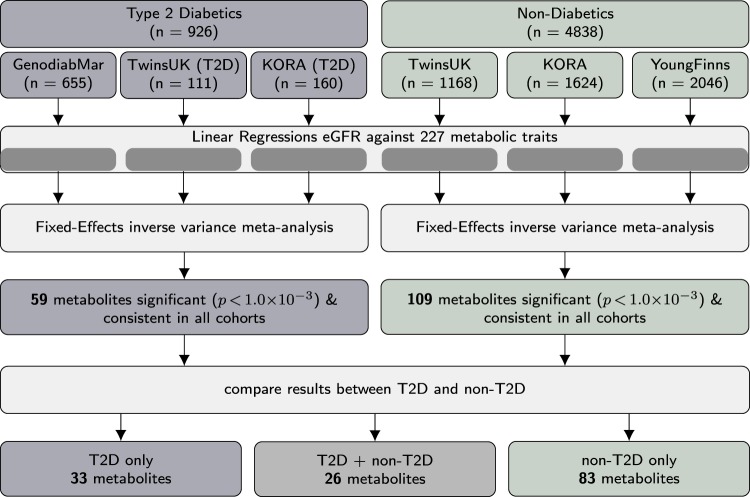
Flowchart illustrating the workflow to identify of metabolic markers of diabetic and non-diabetic renal disease. Associations between circulating metabolites and renal function were assessed in samples from four different cohorts stratified for type 2 diabetes status individually. Results were subsequently meta-analyzed for type 2 diabetics and non-diabetic individuals separately.

Levels of 227 fasting serum metabolic traits including small molecules, lipids, lipoprotein subclasses, their lipids component and fatty acids (Supplementary Table 1), were obtained for 5764 individuals from four independent European cohorts, including 926 T2D patients (Fig. 1). The demographic characteristics of all cohorts are presented in Table 1. We calculated associations of all 227 metabolic traits with renal function in each cohort individually and meta-analyzed results for diabetic and non-diabetic cohorts (Supplementary Table 2). To assess the confounding effect of drug usage, we ran the same models in 1054 individuals from TwinsUK additionally adjusting for statin and hormone replacement therapy (HRT), and in 655 individuals from GenodiabMar adjusting for statin usage. Results remain consistent (Supplementary Table 3).

**Table 1:** General characteristics of the study populations.

	Diabetic cohorts			Non-diabetic cohorts		
	GenodiabMar	TwinsUK (diabetics)	Kora (diabetics)	TwinsUK	Kora (non-diabetic)	YoungFinns
n	655	111	160	1168	1624	2046
Zygosity (MZ/DZ/Single)		30/4/77		466/546/156		
Age (years)	69.70 (±9.32)	68.64 (±8.38)	66.74 (±7.44)	64.83 (±7.91)	60.30 (±8.83)	41.88 (±5.00)
Gender (female)	256 (39.1%)	105 (94.6%)	71 (44.4%)	1118 (95.7%)	845 (52.0%)	1115 (54.5%)
BMI (kg/m^2^)	30.32 (±5.05)	29.33 (±5.55)	31.48 (±5.53)	26.05 (±4.61)	27.82 (±4.58)	26.54 (±5.05)
eGFR (mL/min/1. 73 m^2^)	58.64 (±28.83)	75.80 (±17.64)	76.59 (±18.15)	79.87 (±14.53)	87.80 (±15.38)	94.75 (±12.53)
creatinine (mg/dL)	1.26 (±0.62)	0.84 (±0.24)	0.96 (±0.48)	0.80 (±0.16)	0.83 (±0.23)	0.87 (±0.15)
CKD* (grades 1/2/3/4/5) N and (%)	116/213/202/72/52 (17.7/32.5/30.8/10.9/7.9)	20/76/12/3/0 (18/68.4/10.8/2.7/0)	39/95/24/1/1 (24.3/59.3/15/0.6/0.6)	322/726/118/2/0 (27.5/62.1/10.1/0.1/0)	795/752/73/2/2 (48.9/46.3/4.4/0.1/0.1)	1339/699/7/1/0 (65.4/34.1/0.3/0.04/0)

MZ = monozygotic, DZ = dizygotic. BMI = Body mass index. eGFR = estimated glomerular filtration rate (CKD-EPI equation).

*Grades of Chronic Kidney Disease are stratified per KDIGO recommendation^56^ as: G2 60–89, G3 59–30, G4 15–29 and G5 < 15 mL/min/1.73 m^2^. Values for categorical variables are given as n (percentage); values for continuous variable as mean (±SD).

### Markers of renal function common for diabetics and non-diabetics

**Figure 2:**
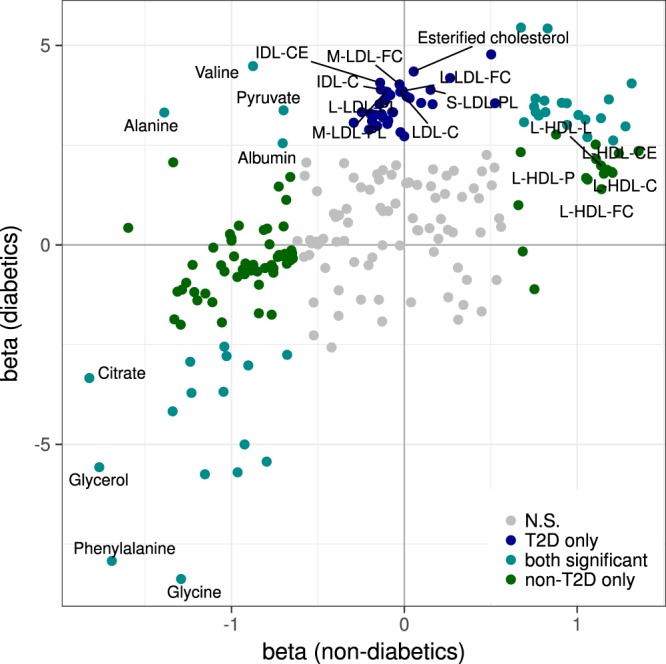
Comparison of metabolic associations with renal function between type 2 diabetics and non-diabetics. We compared associations of metabolic measures with eGFR between diabetic and non-diabetic cohorts. Figure shows effect sizes per 1-SD metabolite concentration from both meta-analyses, colored according to significance level in diabetics (blue), non-diabetics (green) and both (cyan). Non-significant associations are shown in grey (details are shown in Supplementary Table 2).

After adjusting for age, gender, BMI and multiple testing, 26 metabolic traits where consistently associated with renal function across diabetic and non-diabetic cohorts (Table 2). The strongest cross-sectional associations with eGFR were observed for glycine and phenylalanine (P < 0.001) with association magnitudes of −8.37 [−9.73: −7.02] and −7.92 [−9.27: −6.57], respectively, for the diabetic group and −1.29 [−1.66: −0.92] and −1.69 [−2.07: −1.32] for the non-diabetic group (Fig. 2).

**Table 2:** Metabolic traits consistently associated with renal function in diabetic and non-diabetic cohorts.

Class	Trait	diabetics				non-diabetics			
		N	Signs	Beta [95% CI]	p	N	Signs	Beta [95% CI]	p
Amino Acid	Glycine	887	—	−8.37 [−9.73: −7.02]	7.28 × 10^−34^	4632	—	−1.29 [−1.66: −0.92]	6.33 × 10^−12^
	Phenylalanine	905	—	−7.92 [−9.27: −6.57]	1.19 × 10^−30^	4716	—	−1.69 [−2.07: −1.32]	1.11 × 10^−18^
Glycolysis	Citrate	913	—	−3.34 [−4.78: −1.90]	5.68 × 10^−6^	4705	—	−1.82 [−2.18: −1.47]	7.06 × 10^−24^
	Glycerol	551	—	−5.57 [−7.37: −3.77]	1.25 × 10^−9^	3695	—	−1.77 [−2.19: −1.34]	7.54 × 10^−16^
Apolipoproteins	Apolipoprotein A-I	926	+++	3.62 [2.14: 5.10]	1.63 × 10^−6^	4817	+++	0.81 [0.43: 1.19]	3.09 × 10^−5^
Cholesterol	Total cholesterol in HDL2	924	+++	4.05 [2.59: 5.50]	4.94 × 10^−8^	4817	+++	1.32 [0.91: 1.72]	1.51 × 10^−10^
Total cholesterol	Total cholesterol in very large HDL	926	+++	3.18 [1.74: 4.61]	1.41 × 10^−5^	4817	+++	1.14 [0.74: 1.53]	1.62 × 10^−8^
	Total cholesterol in HDL	925	+++	3.65 [2.19: 5.11]	9.65 × 10^−7^	4817	+++	1.18 [0.78: 1.58]	6.55 × 10^−9^
Free cholesterol	Free cholesterol in medium HDL	926	+++	3.47 [2.03: 4.90]	2.22 × 10^−6^	4817	+++	0.75 [0.39: 1.11]	5.25 × 10^−5^
Cholesterol esters	Cholesterol esters in very large HDL	926	+++	3.14 [1.71: 4.57]	1.60 × 10^−5^	4817	+++	1.05 [0.66: 1.44]	1.43 × 10^−7^
Lipoprotein subclasses	Concentration of very large HDL particles	926	+++	2.62 [1.17: 4.07]	4.13 × 10^−4^	4817	+++	1.21 [0.80: 1.62]	5.90 × 10^−9^
	Concentration of medium HDL particles	926	+++	3.33 [1.89: 4.76]	5.41 × 10^−6^	4817	+++	0.76 [0.40: 1.12]	3.27 × 10^−5^
Total Lipids	Total lipids in very large HDL	926	+++	2.97 [1.52: 4.42]	5.71 × 10^−5^	4817	+++	1.28 [0.87: 1.69]	8.36 × 10^−10^
	Total lipids in medium HDL	923	+++	3.33 [1.88: 4.77]	6.29 × 10^−6^	4813	+++	0.82 [0.46: 1.18]	8.81 × 10^−6^
Phospholipids	Phospholipids in medium HDL	926	+++	3.24 [1.80: 4.68]	1.02 × 10^−5^	4817	+++	0.78 [0.42: 1.14]	2.40 × 10^−5^
Total cholesterol (%)	Total cholesterol to total lipids ratio in chylomicrons and extremely large VLDL	739	—	−3.02 [−4.55: −1.49]	1.07 × 10^−4^	4005	—	−0.90 [−1.28: −0.53]	2.21 × 10^−6^
	Total cholesterol to total lipids ratio in IDL	916	+++	5.43 [4.08: 6.77]	2.65 × 10^−15^	4805	+++	0.83 [0.48: 1.18]	3.60 × 10^−6^
Cholesterol esters (%)	Cholesterol esters to total lipids ratio in very small VLDL	920	+++	3.67 [2.28: 5.06]	2.25 × 10^−7^	4808	+++	0.76 [0.40: 1.12]	3.13 × 10^−5^
	Cholesterol esters to total lipids ratio in IDL	917	+++	5.45 [4.10: 6.79]	2.05 × 10^−15^	4807	+++	0.67 [0.32: 1.03]	1.66 × 10^−4^
Triglycerides (%)	Triglycerides to total lipids ratio in very small VLDL	924	—	−3.68 [−5.07: −2.30]	1.93 × 10^−7^	4814	—	−1.05 [−1.42: −0.68]	3.28 × 10^−8^
	Triglycerides to total lipids ratio in large LDL	923	—	−5.70 [−7.04: −4.36]	9.08 × 10^−17^	4813	—	−0.96 [−1.32: −0.61]	7.75 × 10^−8^
	Triglycerides to total lipids ratio in medium LDL	913	—	−5.43 [−6.79: −4.08]	4.05 × 10^−15^	4808	—	−0.80 [−1.15: −0.44]	9.26 × 10^−6^
	Triglycerides to total lipids ratio in small LDL	913	—	−5.00 [−6.37: −3.63]	8.91 × 10^−13^	4807	—	−0.92 [−1.28: −0.57]	3.73 × 10^−7^
	Triglycerides to total lipids ratio in IDL	923	—	−5.75 [−7.10: −4.40]	8.20 × 10^−17^	4811	—	−1.15 [−1.51: −0.80]	2.40 × 10^−10^
	Triglycerides to total lipids ratio in large HDL	886	—	−4.17 [−5.60: −2.75]	9.49 × 10^−9^	4612	—	−1.34 [−1.70: −0.98]	1.71 × 10^−13^
Phospholipids (%)	Phospholipids to total lipids ratio in very small VLDL	923	+++	3.01 [1.61: 4.40]	2.34 × 10^−5^	4808	+++	0.94 [0.58: 1.31]	4.27 × 10^−7^

26 metabolic traits from 14 metabolic classes, listed here, were associated with eGFR consistently across both diabetic and non-diabetic cohorts. Signs represent the directions of the regression coefficients in each diabetic (GenodiabMar, diabetics-TwinsUK, diabetics-KORA) and non-diabetic (TwinsUK, KORA, YoungFinns) cohort. Results were meta-analyzed for diabetic and non-diabetic cohorts separately. Association magnitudes are eGFR per 1-SD (log-transformed) concentration. For detailed list of associations of all 227 analyzed metabolic traits see Supplementary T2.

**Figure 3:**
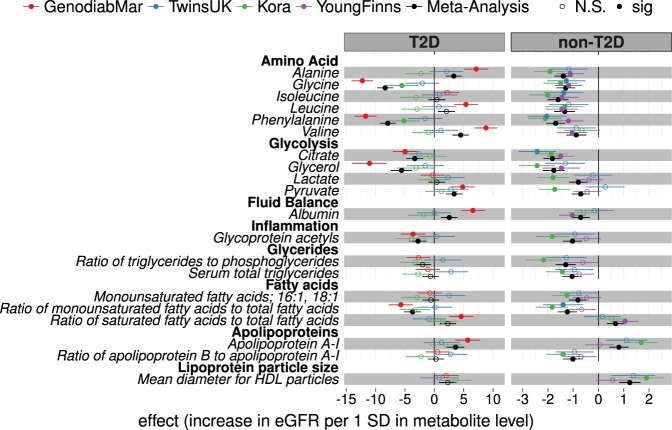
Metabolic traits associated with eGFR in diabetic and non-diabetic cohorts. As for lipoprotein subclasses, associations with eGFR were calculated for several additional metabolic traits. Effect sizes per 1-SD in metabolite concentration and respective 95% confidence intervals are shown for each cohort individually and combined (black). The complete list of results including estimates for cohort heterogeneity can be found in Supplementary Table 2.

Levels of triglycerides in different sizes of intermediate- and low-density lipoprotein (IDL and LDL, respectively) particles were consistently inversely associated with the eGFR, while several high-density lipoprotein (HDL) subclasses of different sizes rich in lipids, cholesterol, cholesterol esters, phospholipids and Apolipoprotein-A1 (Apo-A1) were consistently positively associated with eGFR (Fig. 3).

Citrate and glycerol were consistently negatively correlated with eGFR in all cohorts and also correlated with longitudinal change of eGFR (β[95% CI] = −0.04 [−0.06: −0.02], p = 1.5 × 10^−6^ and −0.02 [−0.04: −0.01], p = 2.6 × 10^−3^) respectively, in TwinsUK and YoungFinns. However, their circulating levels at baseline did not predict eGFR at follow-up in GenodiabMar and diabetic-KORA cohorts (Supplementary Tables 4 and 5).

### Metabolic profiles associated to renal function in diabetics

**Figure 4:**
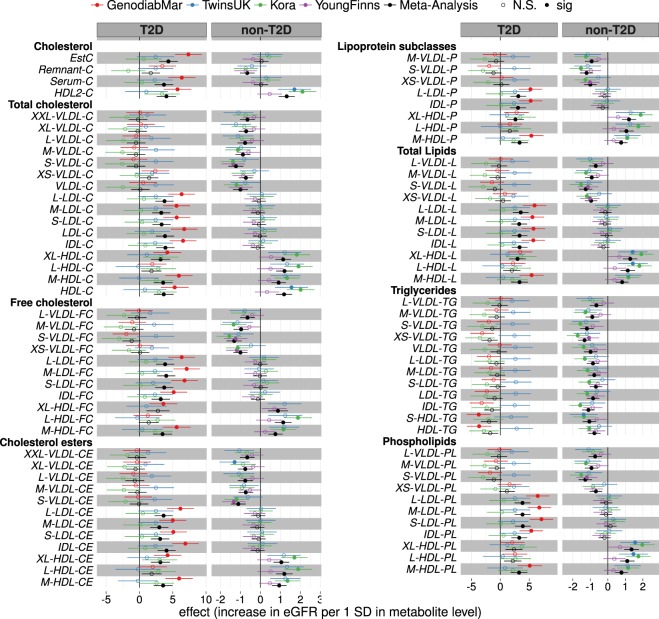
Lipoprotein classes associated with eGFR in diabetic and non-diabetic cohorts. Associations of lipoprotein subclasses with eGFR were calculated in three type 2 diabetic (T2D) and three non-diabetic (non-T2D) cohorts and results were meta-analyzed (black). Here we report regression coefficients and their respective 95% confidence interval per 1-SD (log-transformed) metabolite concentration for each cohort and the meta-analyses. For detailed list of results, including heterogeneity of effect estimates, and full metabolites names see Supplementary Table 2.

In the three diabetic cohorts, 59 metabolic measures were consistently associated with eGFR after meta-analysis at P < 0.001. Of those, 33 traits were associated with eGFR only in diabetics but not in non-diabetics (Supplementary Table 2). 6 of these traits were concentrations of cholesterol esters in LDL and IDL subclasses, 4 phospholipids in LDL and IDL, and 14 cholesterol and lipid concentrations in LDL and IDL that followed a positive association with eGFR in diabetics (Fig. 4 and Supplementary Fig. 1). Also, esterified cholesterol (EC) (β = 4.35 [2.96: 5.74], p = 9.3 × 10^−10^) and total cholesterol (β = 3.68 [2.29: 5.07], p = 2.0 × 10^−7^) were positively associated with eGFR in diabetics only. However, none of this lipoprotein subclasses predicted the change of renal function and only triglycerides to total lipids ratio in large VLDL (β = 0.13 [0.01: 0.25]) and total cholesterol to total lipids ratio in medium VLDL (β = −0.12 [−0.23: 0.00]) were associated to eGFR in the longitudinal analysis (Supplementary Table 5).

**Figure 5:**
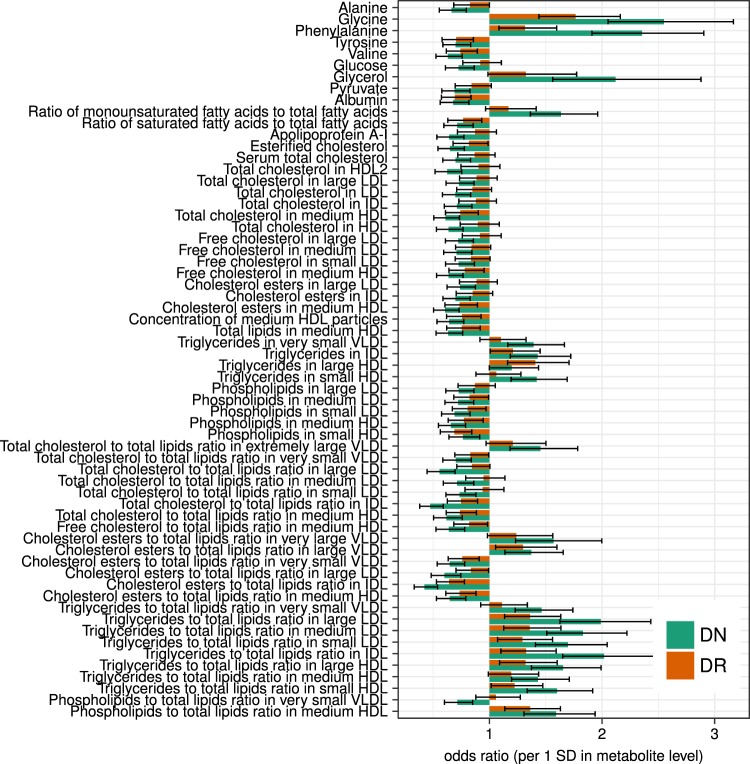
Metabolic measures associated with microvascular complications of diabetes. To further assess associations of metabolic traits with general microvascular damage we compared their association with diabetic nephropathy (DN) and diabetic retinopathy (DR) in the GenodiabMar cohort. Bars represent odds ratios and the respective 95% confidence intervals for each metabolic trait. (For detailed list of results see Supplementary Table 5).

To further explore the relationship of metabolic profiles with other microvascular complications of diabetes, we calculated cross-sectional associations of metabolite profiles with proteinuria (independently of the eGFR), as well as cross-sectional odds-ratios for diabetic nephropathy (DN) and diabetic retinopathy (DR) in GenodiabMar. Glycine and phenylalanine were common risk factors not only for DN but also for both DR and proteinuria (Fig. 5 and Supplementary Table 6). Similarly, pyruvate, which was associated with better renal function, showed an inverse association with proteinuria. Glycerol, citrate, and pyruvate showed concordant albeit non-significant association with the retinal microvascular damage. Moreover, triglyceride contents in IDL, large and medium LDL, and small VLDL were consistently associated with decreased eGFR as well as higher risk of DN and DR, though to a lesser extent. In contrast, many other lipoprotein subclasses, including most HDLs, did not appear to be associated with DR (P > 0.05). As expected serum albumin was strongly associated with better renal function only in GenodiabMar cohort, due to a higher prevalence and a more severe diabetic nephropathy in this population.

### Metabolic profiles associated to renal function in non-diabetics

In the three non-diabetic cohorts 109 metabolic measures were consistently associated with eGFR (P < 0.001) (Supplementary Table 2). 83 of these were associated with renal function only in non-diabetics but not in the diabetic group. Cholesterol and triglyceride levels in VLDL particles of all sizes were negatively associated with eGFR. In contrast, phospholipid in large HDL were positively associated with renal function in non-diabetic populations cross-sectionally (phospholipids to total lipids ratio in very large HDL: β = 0.69 [0.32:1.06], p = 2.8 × 10^−4^) and longitudinally (phospholipids in very large HDL: 0.04 [0.02:0.05], p = 1.3 × 10^−5^) in TwinsUK (Supplementary Tables 2 and 4). Also, this lipid ratio predicted longitudinal change of eGFR independently of baseline eGFR in KORA (0.04 [0.01:0.07], p = 3.7 × 10^−3^) (Supplementary Table 5).

### Discordant metabolic measures between diabetics and non-diabetics

Four metabolites were positively associated with eGFR in diabetics, while they were negatively associated in the non-diabetic individuals at P < 0.001. However, the effect directions of these metabolites were not consistent throughout all cohorts. These include the amino acids alanine (T2D: β = 3.32 [1.92:4.72], non-T2D: −1.39 [−1.75: −1.03]), valine (T2D: 4.48 [3.08: 5.88], non-T2D: −0.88 [−1.27: −0.48]), the glycolysis product pyruvic acid (T2D: 3.37 [1.93: 4.82], non-T2D: −0.70 [−1.06: −0.34]), and albumin (T2D: 2.55 [1.16: 3.93], non-T2D: −0.70 [−1.06: −0.35]) (Supplementary Table 2). Albumin also predicted the longitudinal change of eGFR (0.18 [0.06: 0.30] p = 3.8 × 10^−3^) in the GenodiabMar and KORA cohorts (Supplementary Table 5). Also, there was a trend towards an increase of small, medium, and large LDL particles rich in phospholipids and cholesterol with better eGFR in T2D, that followed an opposite albeit non-significant association in non-diabetic populations (Fig. 4).

## Discussion

In the largest study of its kind, including 926 T2D diabetics and 4838 non-diabetics from four independent European cohorts, we identified 142 metabolic traits consistently associated with renal function at P < 0.001 and with concordant effects across cohorts: 59 in diabetics, 109 in non-diabetics, with an overlap of 26 traits. When comparing the effect directions, associations were largely concordant between diabetic and non-diabetic cohorts (R^2^ = 0.60, Fig. 2). However, there were some notable exceptions. For instance, phospholipids and CE in IDL and LDL were positively correlated with eGFR only in diabetic individuals, while phospholipid content in HDL was positively associated with eGFR both cross-sectionally and longitudinally only in non-diabetics. We additionally identified four traits, valine, alanine, pyruvate, and albumin that were negatively associated with eGFR in non-diabetics, and positively associated in diabetics, though the positive associations were not consistent across all cohorts but driven by the GenodiabMar cohort that has a wider range of renal function impairment.

The metabolic measures identified fall into three categories: amino acids, energy-related metabolites, and lipoprotein subclasses particles and their lipids composition.

### Amino acids

Phenylalanine, serves as precursor for tyrosine in the liver and kidneys^13^. It has been previously associated with insulin resistance, increased risk of T2D^14–17^ and is a predictor of CV events^18^. Moreover, reduced rates of conversion of phenylalanine to tyrosine were observed in CKD^19,20^, leading to decreased circulating levels of tyrosine and increased levels of phenylalanine. While previous studies found the negative association of eGFR with tyrosine stronger than its positive associations with phenylalanine^21^, we found tyrosine levels decreased only in diabetic patients (P < 0.001) Also, the log-fold change of phenylalanine over tyrosine correlated stronger with eGFR in GenodiabMar than either individually (β = 13.13 [11.38:14.88], p = 5.2 × 10^−42^). While increased concentration of phenylalanine was associated with worse renal function in both diabetics and non-diabetics, it was not predictive for disease progression in this study. This suggests that phenylalanine might not be a predictor of renal decline but rather a consequence of renal dysfunction and a maker of vascular damage^18^. Indeed, phenylalanine was also associated at p < 0.05 with DR and albuminuria suggesting an association with endothelial microvascular damage.

Similarly, glycine is converted to serine in the kidneys^22,23^. Thus, impairment of renal function leads to accumulation of glycine, which was consistently observed in both diabetic and non-diabetics. Glycine also correlated with albuminuria and DR at P < 0.001 but did not predict longitudinal change of renal function. Previous studies report glycine to be negatively associated with CV risk factors and T2D^17^, but renal function was not included as a covariate. Also, eGFR usually increases in early stages of diabetes, before renal function declines. Thus, the observed associations with risk for T2D might be confounded by renal function. Our results highlight the importance of including renal function as cofactor when studying diabetes, and are in line with T2D experimental animal model studies, that revealed a lower urine excretion of glycine and accumulation of this metabolite in diabetic kidney tissues^12^.

### Energy-related metabolites

Alanine is a major precursor of hepatic and renal gluconeogenesis and glycolysis via pyruvate pathways. Together with glycerol, which was also negatively associated with eGFR, and glutamine, they constitute 90% of the substrates of gluconeogenesis. Metabolic acidosis induced by CKD leads to increased abundance of circulating alanine, glutamine, and glutamate^23^. However, in the diabetic milieu, glucose metabolism is heavily disturbed with an increased rate of gluconeogenesis^24^. Consequently, the decline of renal function has a different impact on gluconeogenesis in diabetics, as evidenced by the different directions of associations for alanine and pyruvate in this study.

Citrate is an important metabolic substrate in the kidney accounting for up to 10% of the energy production that counteract metabolic acidosis^25^. In agreement with our findings, different studies reported increased concentration with the decline of eGFR^12,26,27^.

### Lipoprotein subclasses and their lipids component

Lipids abnormalities are not always detected in CKD subjects when using standard clinical measures^28,29^. Particularly total and LDL cholesterol are usually normal and even low in advanced CKD^30–33^. The CKD-induced lipid profile has specific characteristics distinct from the general population. Besides quantitative changes, renal patients have several qualitative lipid alterations^34,35^ that cannot be detected by routine determinations and some alterations of the lipoprotein composition and size may contribute to the CV complications observed in CKD patients. Interestingly, in the present study non-classical lipid profiles showed association with renal function and remained associated after adjustment for statin usage (Supplementary Table 2). Some epidemiological studies revealed controversial results regarding lipid-lowering therapy and reduction of cardiovascular mortality in CKD^36–38^ and emphasize the need for further studies such as the present analysis. In our study, the lipid content in the different lipoprotein particles showed considerable differences, which highlights the potential importance of performing a more detailed lipidomic analysis that may reveal different risk patterns that would otherwise be missed.

Some of the largest differences found with renal function between diabetics and non-diabetics were the negative associations of small to large VLDL and LDL subclasses and their respective cholesterol and triglyceride content observed only in non-diabetics, as well as the positive associations of small to large IDL and LDL subclasses and their cholesterol, EC and phospholipid content observed only in diabetics.

The positive association of the pro-atherogenic LDL and IDL with eGFR observed in this study, are likely not reflecting a positive effect of these lipoproteins on renal function but rather a better nutritional status in subjects with better renal function. Higher prevalence of individuals with worse renal function in the T2D cohorts is the likely cause of these counter-intuitive associations^39^. Of note, the phospholipid and CE content of these lipoprotein particles may be related to increased lipid transfer proteins (LTP) activity (CETP and PLTP) present in diabetic subjects^40^. On the contrary, lower activity of LTPs associates with lower CV risk^41^, which might be related to the negative associations of LDL subclasses with renal function in non-diabetic subjects. Interestingly, triglyceride ratios in LDL and IDL were negatively associated with renal function consistently between diabetics and non-diabetics. Also, triglyceride to total lipid ratios showed stronger association in diabetes compared to non-diabetes (Supplementary Table 2). To the best of our knowledge, no studies have investigated the activity of LTP and their association with renal damage and whether pharmacological targeting of this proteins might influence in renal function.

Longitudinal analysis revealed a positive association of several HDL particle and Apo-A1 with renal function over time (Supplementary Table 4). However, their circulating levels at baseline were not associated with a better renal function in T2D at follow-up. Diabetic dyslipidemia presents particularities regarding quantitative lipoprotein abnormalities and also qualitative and kinetic abnormalities that results in a more atherogenic lipid profile^28^. Changes in HDL composition in T2D have been shown to affect cholesterol efflux^42,43^. Moreover, higher proteinuria may increases the loss of particles derived from HDL catabolism^44,45^. In our study, the ratio of phospholipids to total lipids in very-large HDL was associated with longitudinal change of eGFR and predicted future eGFR only in non-diabetics. Phospholipids in HDL enhance its cholesterol efflux capacity^46,47^, which is impaired in diabetics and may explain the observed differences^42,43^.

Although many of our findings are shared with previous studies on T1D^7,8^, we found some differences. For example, we did not find any association of eGFR with sphingomyelin or total fatty acids that were markers of kidney injury and mortality in T1D. This may suggest differences between T1D and T2D metabolic profiles and the importance of analyze both conditions individually.

The present study has several strengths. First, we analyzed data from four independent cohorts, thus minimizing the risk of false positive findings. Second, we analyzed a wide range of metabolic traits beyond those commonly used in clinics. Also, we stratified for diabetes status, thus providing a direct comparison of metabolic profiles associated with diabetic and non-diabetic renal damage. We also note some study limitations. The GenodiabMar cohort was recruited from medical consultations while the other cohorts represent individuals from the general population. Thus, the presence of other medical complications as well as different grades of renal dysfunction may be confounding factors. However, by meta-analyzing results across diabetic cohorts, we controlled for population-specific effects. Also, drug use may have an important impact on metabolic profiles, although statin use did not substantially change the results in this study (Supplementary Table 3). However, further analyses specifically addressing the effects of different drugs, such as antihypertensive, other medical conditions and the potential effect of renal replacement therapies, are needed.

In conclusion, we found widespread metabolic changes associated with decline of renal function. While associations of many lipoprotein particles and their lipid composition with renal function were largely similar between diabetic and non-diabetic cohorts, several exceptions revealed metabolic differences between the conditions. Also, changes of amino acid and energy metabolism were markedly different regarding diabetes condition. Our results show alterations of lipoprotein composition in kidney disease that are currently underexploited in clinics. We also find marked metabolic differences between diabetic and non-diabetic kidney disease, suggesting that more specific markers for each condition might be able to outperform current markers of kidney disease.

## Methods

### Study Design and Participants

Targeted NMR metabolic profiling was conducted in 926 diabetic and 4838 non-diabetic individuals from the GenodiabMar (n = 655), TwinsUK (n = 1279, 111 with T2D)^48^, KORA (n = 1784, 160 with T2D)^49^, and Young Finns (n = 2046)^50^ cohorts. GenodiabMar is a cohort of T2D patients, recruited in a hospital, while the other cohorts were recruited from the general population. Renal function was measured as eGFR from standard creatinine using the Chronic Kidney Disease Epidemiology Collaboration equation (CKD-EPI)^51^. Longitudinal measures were available for a subset of 3644 individuals (Supplementary methods). Each local ethics committee approved the study, and subjects were included after providing informed consent. All methods were performed in accordance with the relevant guidelines and regulations.

A flowchart of the study design is depicted in Fig. 1.

### Metabolic profiling

Metabolic profiling of 227 metabolic traits, 143 metabolite concentrations, 80 lipid ratios, 3 lipoprotein particle sizes and a semi-quantitative measure of albumin (see Supplementary Table 1 for full list), was conducted for all cohorts by Nightingale Health Ltd. (Helsinki, Finland; previously known as Brainshake Ltd) using a targeted NMR spectroscopy platform that has been extensively applied for biomarker profiling in epidemiological studies as previously described^18,52,53^ (Supplementary methods).

### Statistical analysis

All metabolic measures were log-transformed. To account for zero values a pseudo-count of 1 was added to all measurements prior to transformation. All measurements were shifted to zero mean and scaled to standard deviation (SD) of 1 (z scores) to facilitate comparisons across cohorts. The average absolute concentrations and SDs of each metabolite in each cohort are presented in Supplementary Table 1.

#### Cross-sectional analysis

We assessed the associations between metabolic profiles and renal function in each cohort individually by fitting linear regressions for all metabolic traits with eGFR as outcome, adjusting for age, gender, BMI (and family relatedness as random intercept) to account for the decline of renal function with advancing age as well as its dependency on obesity. All results were then meta-analyzed separately for T2D patients and non-diabetic cohorts using inverse variance fixed effect meta-analysis due to the expected homogeneity of effects in both subgroups. We adjusted for multiple testing using Bonferroni correction assuming 50 independent test as suggested by Li and Ji^54^ (P < 0.001) (Supplementary methods).

As metabolic profiles may be strongly affected by medication such as statin treatment^55^ or hormone replacement therapy, we tested the robustness of our results by running a sub-analysis in a subset of individuals from TwinsUK and GenodiabMar with information on treatment available, additionally adjusting for medication status.

To further investigate traits of interest, we regressed the concentration of albumin in urine against each of the metabolic traits. Finally, we calculated logistic regression models to assess the association of each metabolic trait with diabetic nephropathy and retinopathy, respectively.

#### Longitudinal analysis

For TwinsUK and YoungFinns we estimated the trajectories of metabolite/eGFR change by fitting linear mixed models for each metabolite with a per-individual random effect for the time since baseline. The estimate of this random effect provides a measure of the (linear) change of metabolite concentration over time similarly to calculating the change per year for two visits. These trajectories were estimated for all metabolites individually and then compared to the change in eGFR in a separate regression model, thus assessing longitudinal correlations between metabolites and renal function.

Also, we evaluated the potential of metabolite measures as diagnostic tool by predicting the eGFR at follow-up using metabolic measures at baseline, correcting for gender and baseline eGFR, age, and BMI.

## Electronic supplementary material

Supplementary Information

Supplementary Tables

## Data Availability

Data from the TwinsUK cohort are available upon request on the department website (http://www.twinsuk.ac.uk/data-access/accessmanagement/). Data from the KORA cohort can be requested online (https://epi.helmholtz-muenchen.de/) and is subject to approval by the KORA board.
