# Supplementary material for: Circulating metabolic biomarkers of renal function in diabetic and non-diabetic populations
Source: Sci Rep. 2018 Oct 15;8:15249. doi: 10.1038/s41598-018-33507-7 (PMC6189123; doi:10.1038/s41598-018-33507-7)
Supplement: Supplementary file 1 — Supplementary Information [file 41598_2018_33507_MOESM1_ESM.docx]

**Circulating metabolic biomarkers of renal function in diabetic and non-diabetic populations**

Clara Barrios^1,2*^, Jonas Zierer^1,3*^, Peter Würtz^4,5^, Toomas Haller^6^, Andres Metspalu^6^, Christian Gieger^7,8^, Barbara Thorand^7^, Christine Meisinger^7,9^, Melanie Waldenberger^7,8^, Olli Raitakari^10,11^, Terho Lehtimäki^12^, Sol Otero^2,13^, Eva Rodríguez^2^, Juan Pedro-Botet^14^, Mika Kähönen^15^, Mika Ala-Korpela^16,17,18^, Gabi Kastenmüller^3^, Tim D. Spector^1^, Julio Pascual^2**^, Cristina Menni^1**^

# Supplementary Methods

## Participants and Phenotypes

### GenodiabMar

The GenodiabMar study is a T2D adult registry, recruited between 2012 and 2015 from the healthcare area Litoral-Mar of Barcelona, Spain, to investigate microvascular complications of diabetes. Patients older than 45 with a medical history of T2D for more than 10 years and under anti-diabetic drug therapy were included. Renal ultrasound, fundoscopy, and measurements of proteinuria were performed for all participants. For the present study, we analyzed 655 individuals with NMR metabolomic data available. Longitudinal measurements of creatinine were available for a subset of 326 individuals with an average follow up of 4±1.5 years. 20 cc Ethylene diamine tetra acetic (EDTA) fasting blood sample was taken from all participants. Samples were centrifuged (2500rpm, 10 min) and stored at -80ºC until used. In participants under hemodialysis treatment (n=49), fasting samples were taken before the procedure of the mid-week treatment. All samples were sent to Nightingale Inc. for metabolomics analyses in a single batch. The study was approved by the ethics committee of Institut Hospital del Mar d'Investigacions Mèdiques, Barcelona, Spain.

### TwinsUK

TwinsUK is a national register of twins recruited through media campaigns and representative of the general population in terms of lifestyle parameters^1^. All participants are regularly invited to visits at St. Thomas Hospital, London, UK, for clinical assessment as well as sample collection. Fasting blood samples were collected during visits, aliquoted, and stored at -80 ºC until analysis. For this study, we included 1279 individuals (111 with T2D) with metabolomic data and clinical creatinine available. Also, longitudinal measures of clinical creatinine and metabolomics were available for 740 individuals, 363 of them examined at three time points, with an average follow-up time of 11.8±4.7 years. Metabolomics was measured by Nightingale Inc. in two batches. The study was approved by the ethics committee of St. Thomas Hospital, London, UK.

### KORA

The Cooperative Health Research in the Region of Augsburg (KORA) study is a group of subsequent population cohorts based in the south of Germany^2^. For the most recent cohort, S4, 4261 individuals were initially recruited in 1999. 3080 of those were recalled between 2006 and 2009 for the F4 follow up and 2279 were recalled a second time in 2014 for the FF4 follow up. 1784 (160 with T2D) participants of the F4 recall for which metabolomics and clinical creatinine measurements are available, were included here. Follow-up measurements of creatinine were available from the FF4 recall for a subset of 1185 individuals, 7 years after the baseline visit. Blood samples were drawn during the participant’ visit to the study center between 8:00 and 10:30am after at least 10 hours of overnight fasting and kept at 80 ºC until further analysis^3^. All samples were sent to Nightingale Inc. for metabolomics analyses in a single batch. ﻿The study was approved by the Ethics Committee of the Bavarian Medical Association.

### YoungFinns

Additionally, data from 2046 individuals from the YoungFinns cohort were analyzed. The ‘Cardiovascular Risk in Young Finns’ study was started as a pilot in 1978 and the first baseline, incorporating 3596 children aged 3 to 18 years was recruited in 1980. After that follow-ups were conducted approximately every three years^4^. Blood samples were drawn at the study center after overnight fasting. Here, we analyzed data from the 21, 27, and 30-year follow-ups, for which blood metabolomics data was available. Longitudinal measurements of creatinine and metabolomics at all three time points were available for 1770 of the 2046 individuals. The study data collection has been approved by the hospital district of Southwest Finland ethics committee.

## Phenotypes

For all cohorts, renal function was measured as eGFR from standard creatinine, calculated using the Chronic Kidney Disease Epidemiology Collaboration equation (CKD-EPI)^5^. The grades of renal disease were defined by the current QDIGO guidelines^6^. Age, gender, and body mass index (BMI) were additionally collected as confounding factors. Data on usage of statins and hormone replacement therapy (HRT) were available for a subset of individuals. For the GenodiabMar cohort, diabetic retinopathy was diagnosed by an ophthalmologist and proteinuria, was obtained as protein-to-creatinine ratio (in mg/g) in three independent spot urine samples.

Diabetic nephropathy (DN) was defined as eGFR<=60ml/min/1.73m^2^ and/or proteinuria >=300mg/g, or proteinuria 30-299mg/g and diabetic retinopathy (DR). Analyses were stratified by T2D status. As previously described, all participants of the GenodiabMar cohort were diagnosed of T2D by a medical doctor. In TwinsUK and KORA 111 and 160 participants, respectively, reported to be diabetic and were, thus, analyzed separately.

## Metabolic profiling

Metabolic profiling was conducted for all cohorts by Nightingale Health Ltd. (Helsinki, Finland; previously known as Brainshake Ltd) using a targeted NMR spectroscopy platform, as previously described^7^. Briefly, samples are mixed with sodium phosphate buffer and subsequently transferred to SampleJet ^1^H NMR tubes (Bruker, Billerica, MA, USA) using a PerkinElmer JANUS handler (Waltham, MA, USA). Samples are analyzed on a Bruker AVANCE III (Bruker, Billerica, MA, USA) 500 MHz spectrometer for 5 min. Two control samples, one plasma sample and one mixture of two low-molecular weight metabolites, are added to each 96-well plate for quality control. The initial data processing, including the Fourier transformations to NMR spectra and automated phasing are done using the computers that control the spectrometers; the spectra are then automatically transferred to a centralized server, which performs various further automated spectral processing steps, including overall signal check for missing/extra peaks, background control, baseline removal and spectral area-specific signal alignments. The spectral information of the actual sample also undergoes various comparisons with the spectra of the 2 quality control samples; the data for which is also followed and compared in a consecutive manner. For those spectral areas that pass all the quality control steps, regression modeling is performed to produce the quantified molecular data. A proprietary Bayesian algorithm^8^ is used to quantify absolute concentrations of a predefined set of 144 metabolic traits, including 98 lipid constituent measures from 14 lipoprotein subclasses as well as 9 amino acids, from the NMR spectra. Moreover, the algorithm provides measures of average particle sizes for very-low-density lipoprotein (VLDL), low-density lipoprotein (LDL), intermediate-density lipoprotein (IDL), and high-density lipoprotein (HDL) as well as a semi-quantitative measure of albumin concentration. Additionally, 80 ratios of metabolic measures describing the composition of lipoprotein particles were analyzed, totaling to 227 metabolic traits (**Supplementary Table 1**). The NMR platform has been extensively applied for biomarker profiling in epidemiological studies as reviewed elsewhere^7,9^.

## Statistical analysis

All metabolic measures were log-transformed. To account for zero values a pseudo-count of 1 was added to all measurements prior to transformation. Outliers, differing more than 6 standard deviations (SDs) from the population mean, were excluded. Prior to analysis all measurements were scaled to SD of 1 to facilitate comparisons across cohorts.

### Cross-sectional analyses

First, we assessed the associations between metabolic profiles and renal function in each cohort by fitting linear regression models for all metabolic traits with eGFR as outcome, adjusting for age, gender, and BMI. Regression models were additionally adjusted for family structure by adding a family-wise random intercept in the TwinsUK cohort. Next, results were meta-analyzed separately for T2D patients and non-diabetic cohorts using inverse variance fixed effect meta-analysis as implemented in the R package meta. Moreover, we additionally adjusted the models for statin usage and hormone replacement therapy in a subset of the TwinsUK and GenodiabMar cohorts with data on drug usage available.

As the lipid subclasses are very highly correlated, we estimated the number of effective independent variables *M_eff_* for each cohort using the eigenvalues of the correlation matrix^10^, as

$M_{eff}= \sum_{i=1} f\left( \left| \lambda_{i} \right| \right)$ with $f\left( x \right)=I\left( x\geq1 \right)+(x- \left\lfloor x \right\rfloor)$

Where *λi* is the *i*-th of n eigenvectors and *I(a)* is the indicator functions returning 1 if *a* is true. The highest estimate for the number of independent variables was 49.5 (GenodiabMar cohort). Thus, all analyzed were adjusted for 50 independent tests using Bonferroni correction (p<1.0×10^−3^ = 0.05/50). Only metabolic traits passing the Bonferroni threshold after meta-analysis and with consistent effect directions in all cohorts were considered significant.

To further analyses associations with other microvascular complications of diabetes, we calculated logistic regression models for DN and DR as well as linear regression models regressing proteinuria against each of the metabolic traits.

### Longitudinal analyses

Longitudinal follow-up measures for both clinical creatinine and NMR metabolomics were available for TwinsUK and YoungFinns cohorts. We calculated the longitudinal trajectories of eGFR and each of the metabolites over time by fitting linear mixed models, regressing each of these variables against the time since baseline as per-individual random effect using the lmer function implemented in the R package lme4 (version 1.1)^11^. The estimates of per-individual random slopes of these models were used as estimates of the longitudinal trajectories. Then, the trajectory of eGFR was regressed against each of the metabolite trajectories to analyze longitudinally correlations.

A follow-up for clinical creatinine was available for subsets of 326 individuals from the GenodiabMar cohort and for 1185 individuals from the KORA cohort. We evaluated the potential of metabolite levels as diagnostic tool by predicting the eGFR at follow-up using metabolic measures at baseline, correcting for gender and baseline eGFR, age, and BMI.

## References

1. Moayyeri, A., Hammond, C. J., Valdes, A. M. & Spector, T. D. Cohort Profile: TwinsUK and Healthy Ageing Twin Study. *Int. J. Epidemiol.* **42,** 76–85 (2013).

2. Holle, R., Happich, M., Löwel, H. & Wichmann, H. KORA - A Research Platform for Population Based Health Research. *Gesundheitswesen* **67,** 19–25 (2005).

3. Shin, S.-Y. *et al.* An atlas of genetic influences on human blood metabolites. *Nat. Genet.* **46,** 543–50 (2014).

4. Raitakari, O. T. *et al.* Cohort profile: The cardiovascular risk in young Finns study. *Int. J. Epidemiol.* **37,** 1220–1226 (2008).

5. Levey, A. S. *et al.* A new equation to estimate glomerular filtration rate. *Ann. Intern. Med.* **150,** 604–612 (2009).

6. Stevens, P. E., Levin, A. & Kidney Disease: Improving Global Outcomes Chronic Kidney Disease Guideline Development Work Group Members. Evaluation and management of chronic kidney disease: synopsis of the kidney disease: improving global outcomes 2012 clinical practice guideline. *Ann. Intern. Med.* **158,** 825–30 (2013).

7. Soininen, P., Kangas, A. J., Würtz, P., Suna, T. & Ala-Korpela, M. Quantitative Serum Nuclear Magnetic Resonance Metabolomics in Cardiovascular Epidemiology and Genetics. *Circ. Cardiovasc. Genet.* **8,** 192–206 (2015).

8. Vehtari, A. *et al.* A novel Bayesian approach to quantify clinical variables and to determine their spectroscopic counterparts in 1H NMR metabonomic data. *BMC Bioinformatics* **8 Suppl 2,** S8 (2007).

9. Würtz, P. *et al.* Quantitative Serum NMR Metabolomics in Large-Scale Epidemiology: A Primer on -Omic Technology. *Am. J. Epidemiol.* (2017). doi:10.1093/aje/kwx016

10. Li, J. & Ji, L. Adjusting multiple testing in multilocus analyses using the eigenvalues of a correlation matrix. *Heredity (Edinb).* **95,** 221–227 (2005).

11. Bates, D., Mächler, M., Bolker, B. & Walker, S. Fitting Linear Mixed-Effects Models Using lme4. *J. Stat. Softw.* **67,** 51 (2015).

# Supplementary Figures

## Supplementary Figure S1: Lipoprotein ratios associated with eGFR in diabetic and non-diabetic cohorts.

Associations of lipoprotein ratios with eGFR were calculated in three type 2 diabetic (T2D) and three non-diabetic (non-T2D) cohorts and results were meta-analyzed (black). Here we report regression coefficients and their respective 95% confidence interval per 1-SD metabolite ratio for each cohort and the meta-analyses. For detailed list of results and full metabolites names see **Supplementary T2**.


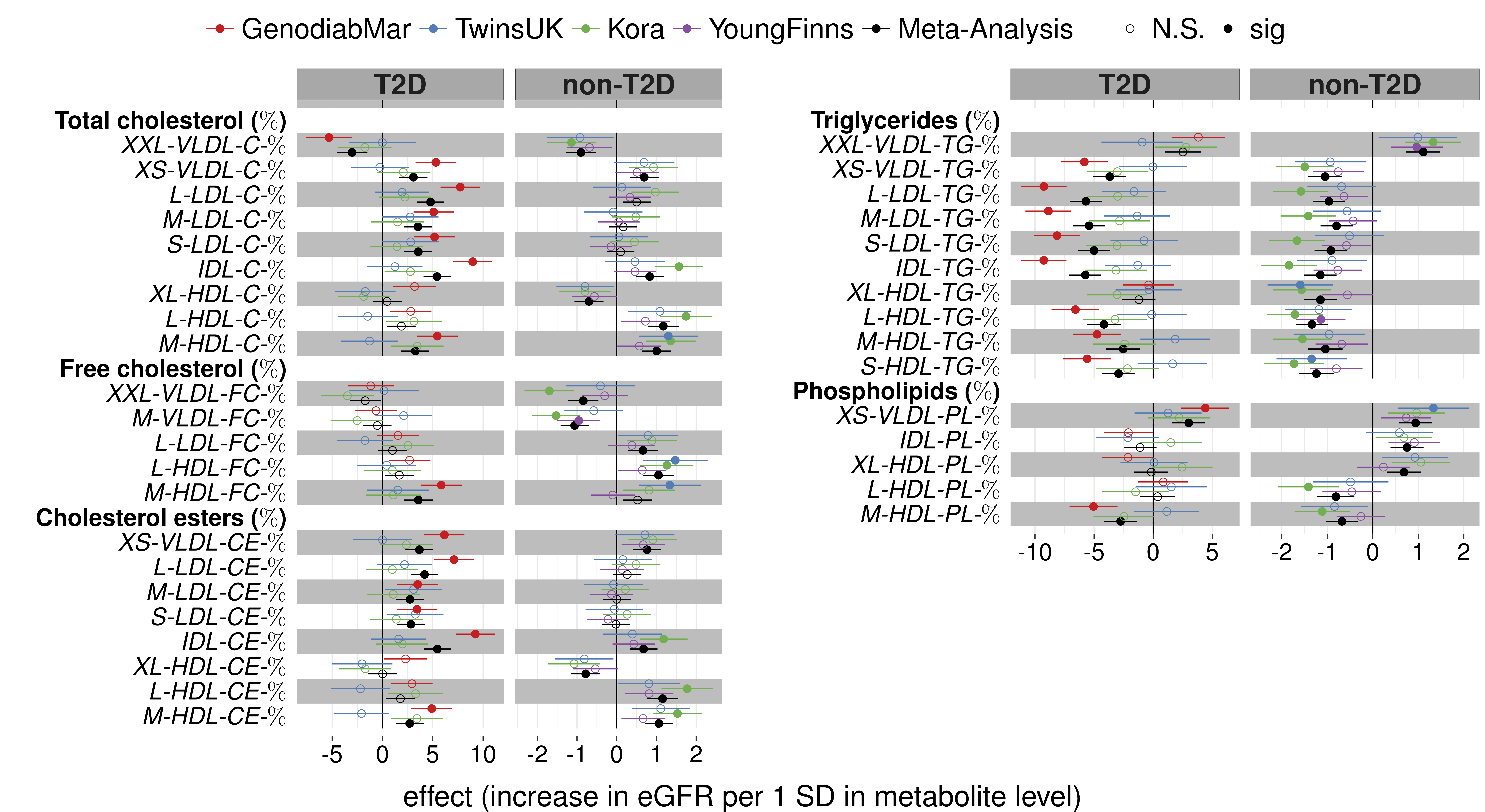


# Supplementary Tables

## Supplementary Table 1: Absolute metabolite concentrations

To facilitate comparability between cohorts we transformed metabolite levels to z scores. Here, we report the average absolute levels and standard deviations of each metabolite per cohort.

## Supplementary Table 2: Metabolic traits associations with eGFR.

Association of 227 metabolic traits with eGFR in four different cohorts were analyzed, stratifying by type 2 diabetes status. Significantly associated metabolite measures (meta-analysis p-value < 10e^-3^ and consistent direction of effect in all cohorts) are indicated in green. Metabolic traits are separated in four groups (first column): (1) associated in diabetics and non-diabetics, (2) associated only in diabetics with no association in non-diabetics (p>0.05 in all non-diabetic cohorts), (3) associated only in non-diabetics (p>0.05 in all diabetic cohorts), (4) metabolites significantly associated in diabetics and non-diabetics with opposite directions of effects (though these traits are not consistent across the cohorts).

## Supplementary Table 3: Longitudinal associations

In the TwinsUK and YoungFinns cohorts longitudinal measurements of both NMR measures and creatinine were available. Trajectories of metabolite and eGFR change were estimated by linear mixed models and then associated, correcting for baseline age, BMI and gender.

## Supplementary Table 4: Prediction of eGFR

Longitudinal follow-ups of clinical creatinine were available in GenodiabMar and KORA. We predicted the future eGFR based on baseline metabolic measures, eGFR, age, BMI and sex.

## Supplementary Table 5: Metabolic associations with microvascular damage

We further investigated associations of metabolites with other microvascular complications of diabetes, diabetic nephropathy, diabetic retinopathy, and albuminuria, in the GenodiabMar cohort, correcting for the same covariates as before. Bonferroni-significant associations are indicated in green.

## Supplementary Table 6: Metabolic associations with eGFR, correcting for drug usage

To assess the potential confounding of our results by drug usage, we calculated the same models as before (Supplementary T1) for 1054 individuals of the TwinsUK cohort additionally adjusting for statin and hormone replacement therapy (HRT), and for the GenodiabMar cohort adjusting for statin usage.
